# Effects of nebulized dexamethasone on the respiratory microbiota and mycobiota and relative equine herpesvirus‐1, 2, 4, 5 in an equine model of asthma

**DOI:** 10.1111/jvim.15671

**Published:** 2019-12-03

**Authors:** Stephanie L. Bond, Matthew Workentine, Jana Hundt, UCVM Class of 2019, James R. Gilkerson, Renaud Léguillette

**Affiliations:** ^1^ Faculty of Veterinary Medicine University of Calgary Calgary Alberta Canada; ^2^ Centre for Equine Infectious Disease, Melbourne Veterinary School The University of Melbourne Melbourne Victoria Australia

**Keywords:** equine herpesvirus, mild equine asthma, qPCR, respirable dust, REST analysis

## Abstract

**Background:**

Prolonged exposure to environmental antigens or allergens elicits an immune response in both healthy horses and those with mild asthma. Corticosteroids often are used to treat lower airway inflammation.

**Objective:**

To investigate the changes in equine herpesvirus (EHV)‐1,2,4,5 glycoprotein B gene expression and changes in respiratory bacterial and fungal communities after nebulized dexamethasone treatment of horses with asthma.

**Animals:**

Horses with naturally occurring mild asthma (n = 16) and healthy control horses (n = 4).

**Methods:**

Prospective, randomized, controlled, blinded clinical trial. Polymerase chain reaction amplification of EHV‐1,2,4,5 in bronchoalveolar lavage fluid, and 16S (microbiome) and ITS2 (mycobiome) genes with subsequent sequencing was performed on DNA extracted from nasal swabs and transendoscopic tracheal aspirates before and after 13 days treatment with nebulized dexamethasone (15 mg q24h) and saline (control).

**Results:**

Nebulized dexamethasone treatment decreased microbial diversity; relative abundance of 8 genera in the upper respiratory tract were altered. For both the microbiota and the mycobiota, environment had a dominant effect over treatment. *Alternaria*, an opportunistic pathogen and allergen in humans recognized as a risk factor for asthma, asthma severity, and exacerbations, was increased with treatment. Treatment affected relative quantification of the equine gamma herpesviruses (EHV‐2 and ‐5); EHV‐2 DNA levels increased and those of EHV‐5 decreased.

**Conclusions:**

Nebulized dexamethasone treatment affected the upper respiratory tract microbiota, but not the mycobiota, which was overwhelmed by the effect of a sustained dusty environment.

**Abbreviations**
EHV: equine herpesvirus
BALF: bronchoalveolar lavage fluid
OTU: operational taxonomic unit
PCoA: principal coordinates analysis

## 1 INTRODUCTION

Mild asthma in horses is a commonly occurring inflammatory airway disease,^1^ which provides a biologically appropriate model for allergic and nonallergic phenotypes of asthma in humans.^2^ It is a naturally occurring disease that, similar to asthma in humans, likely is triggered by a combination of pathophysiological mechanisms. Interactions between environmental factors and immunological defects possibly could be central to the development of an exaggerated lower airway inflammatory immune response. Exposure to an allergenic environment for a prolonged period elicits an immune response in the respiratory tract of both healthy and asthmatic horses and humans, which appears to be dose‐dependent.^3^, ^4^, ^5^, ^6^, ^7^ Adaptations to the local bacterial, fungal, and viral communities of the respiratory tract could provide key insights into asthma phenotype expression. It is as yet unknown whether these adaptations reflect the cause or effect of lower airway inflammation observed in asthma.

The large physical size and relative ease of respiratory sample collection in horses constitutes a clear benefit when compared to the invasive nature of performing these tests on humans.^8^ Furthermore, inclusion of appropriate, untreated negative control groups that are an important ethical difficulty in studies of humans, can be included in asthma studies of horses. This permits investigation into the local environment in the lungs, as well evaluating translocation of microbial flora and interactions between the upper and lower respiratory tract. Although the pathophysiologic mechanisms behind the disease have not been fully elucidated, it is widely thought to be a multifactorial disease, with environmental factors central to the development of lower airway inflammation.^1^ Large amounts of airborne particulates including dust, endotoxin, fungi, molds, ultrafine particles, and noxious gases are found in conventional horse stables,^9^ and there is strong evidence that stabling of horses is a risk factor for mild asthma.^10^, ^11^, ^12^, ^13^ Furthermore, a study investigating biomarkers involved in the development of airway disease reported that some employees involved in the care and training of horses have signs of bronchial obstruction, which might be provoked by working in a stable environment.^14^

However, veterinary recommendations regarding changes to environmental management practices in an effort to limit dust exposure often receive poor owner compliance because of challenges with implementation, and medical treatment, primarily corticosteroids to decrease airway inflammation is the preferred option for many clients.^15^

Although viral infection is a common cause of transient lower airway inflammation in horses, the role of viral infection in the pathogenesis of mild asthma in horses remains controversial.^16^, ^17^, ^18^ Viral respiratory tract infections have a marked effect on the expression of asthma, as well as disease exacerbation, and are a major cause of morbidity.^19^ Although acute infection with viruses including equine herpesvirus (EHV)‐1 and EHV‐4 can be subclinical or cause mild clinical signs consistent with mild asthma, infections usually are self‐limiting.^20^ Although EHV‐2 and EHV‐5 are ubiquitous in healthy horses,^21^, ^22^, ^23^ they frequently are identified in respiratory secretions of horses with respiratory disease,^17^, ^21^ and some evidence indicates that infection with EHV‐2 is associated with poor performance and airway inflammation.^17^ A frequent observation made after cessation of treatment for mild asthma in horses is the reemergence of clinical signs. Given the ubiquitous nature of EHV, as well as their ability to induce latent infection and recrudesce in times of immunosuppression or stress, it is logical to question their role in mild asthma of horses.

Recently, it has been shown that the nebulization of dexamethasone does not induce airway inflammation and has minimal systemic bioavailability.^24^ This represents an inexpensive method to deliver a corticosteroid directly to the lungs, with the intent to minimize possible adverse systemic effects. Because owner compliance with environmental improvements often is poor, our objective was to investigate the effects of nebulized dexamethasone on relative EHV‐1,2,4,5 expression in bronchoalveolar lavage (BAL)‐derived cells, and on the local bacterial and fungal communities of the respiratory tract while housing horses in indoor stables and feeding them from hay nets.

## 2 MATERIALS AND METHODS

### 2.1 Animal care statement

This study was conducted in accordance with the recommendations of the Canadian Council of Animal Care. The research protocol was reviewed and approved by the University of Calgary Veterinary Sciences Animal Care Committee (AC17‐0097).

### 2.2 Animals and study design

**Figure 1:**
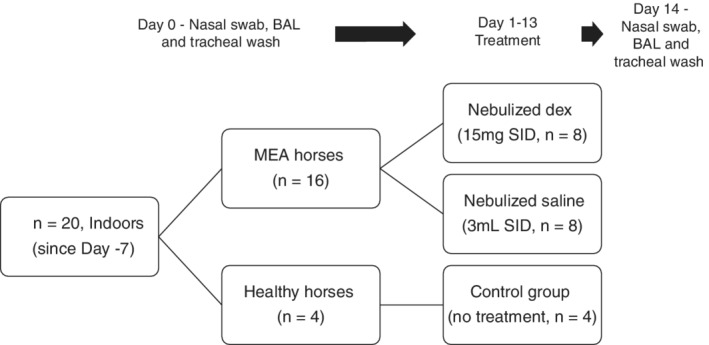
Representation of protocol and treatment group allocation. Horses (n = 20) were allocated into two groups on the basis of their bronchoalveolar lavage (BAL); healthy horses (n = 4) with a normal BAL, and horses with mild equine asthma (MEA, n = 16). Dex, dexamethasone

This study was a prospective, randomized, controlled, blinded clinical trial. Gelding horses (n = 20, 435‐612 kg) were enrolled in the study based on an external veterinarian's diagnosis of mild asthma. Horses had a history of nasal mucus and coughing. Inclusion criteria during this screening stage were a physical examination within normal limits, aside from nasal mucus and coughing, and no signs of systemic illness on CBC or serum biochemistry analysis. Horses were vaccinated regularly with 3‐way core vaccines only (no equine influenza or EHV); they received a core vaccination booster approximately 4 weeks before entering the facilities for the study. Horses resided on 2 properties (Lake Louise and Cochrane, AB, Canada) and were transferred to an indoor stable on day 7. Individual stalls with open tops, enabling free movement of air among stalls, with straw bedding were used. Horses were fed grass hay suspended in hay nets for the duration of the trial and were given free access to water. Horses were lightly exercised every second day, and cough scoring was performed during exercise and stall cleaning (data not shown). Respirable dust concentrations were measured for 4‐minute sampling periods every 4 hours throughout the trial (SidePak AM520, TSI, Minnesota) using a PM_4_ impactor (4 μm); the flow rate (1.7 L/min) was verified using a flow calibrator (4140 Flowmeter, TSI). The air sampler was centrally located within the stable, 1.8 m above the ground. Dust concentration data were analyzed using commercially available software (TrakPro 5, TSI). Nasal swabs, transendoscopic tracheal washes, and BAL were performed on all horses (n = 20) on day 0 (Figure 1). On day 1, horses were allocated into 1 of 3 treatment groups (A, B, C) based on their disease status (see below for inclusion criteria; asthmatic versus healthy horses) and random selection (within horses with asthma; Figure 1). Horses were considered to have asthma based on the following inclusion criteria: (1) inflammatory BAL results with an increased percentage of mast cells (>2%), eosinophils (>0.5%), neutrophils (>5%), or some combination of these; (2) history of nasal mucus, coughing or both, confirmed during clinical examination; and (3) absence of labored breathing at rest.^1^ Group A was treated with 15 mg (5 mg/mL) nebulized dexamethasone sodium phosphate q24h (horses with asthma; n = 8), group B was treated with 3 mL of nebulized saline q24h (horses with asthma; n = 8), and group C was a no‐treatment environmental control (healthy horses; n = 4; Figure 1). All horses were treated for 13 days, and the nasal swab, transendoscopic tracheal wash, and BAL procedures were repeated on day 14 (Figure 1). The nebulizer was disinfected using a standard operating protocol between each horse (available upon request). No other medications were given to horses for the duration of the trial. Those administering treatments were blinded to the treatment provided to groups A and B, as were the specialists who reported the BAL results and those dealing with laboratory investigations.

### 2.3 Sampling procedures

Horses were premedicated with acepromazine maleate (0.07‐0.08 mg/kg, IM or IV), and sedated with xylazine hydrochloride (0.4‐0.5 mg/kg, IV) and butorphanol tartrate (0.05‐0.1 mg/kg). After sedation, nasal swab samples were collected first and then transtracheal samples were collected before BAL.

Nasal swab samples were collected using 15.2 cm sterile flocked Nylon swabs (ESwab, Copan Diagnostics, Murrieta, California) and stored in sterile tubes without transport medium. Two nasal swabs were obtained per horse (1 per nasal cavity). Control swabs also were collected on each collection day (n = 2) with the tip of the swab being exposed to the stable air. Immediately after collection, nasal swabs were frozen at −20°C and transferred within 4 hours to −80°C storage pending DNA extraction.

A transendoscopic tracheal wash then was performed as previously described^25^ with the following modifications: A self‐manufactured sterilized plastic catheter was introduced into the sterilized biopsy channel of a 1.3 m videoendoscope (GIF‐130, Olympus, Canada) until 2‐3 cm emerged from the distal end. Sterile saline (approximately 3 mL) was injected through the catheter into a sterile 10 mL plain tube and stored at −20°C, to be used as a negative control. The tubing then was retracted until shielded in the endoscope; the endoscope then was introduced into the ventral meatus, passed into the trachea, and advanced to approximately 90 cm from the nares. The catheter then was advanced until observed to be protruding from the distal end of the endoscope, and the walls of the tracheal lumen were lavaged with approximately 10 mL sterile saline; the fluid then was aspirated. The aspirate was immediately transferred into a sterile 10 mL plain tube and stored at −20°C; samples were transferred within 4 hours and stored at −80°C pending DNA extraction.

A BAL then was performed as previously described.^26^ Briefly, a balloon‐tipped BAL tube (Mila International, SKU: BAL300) was inserted until wedged against the wall of a bronchus, and 2 boluses (250 mL/bolus) of sterile isotonic saline (0.9% NaCl) solution were instilled sequentially. Lavage fluid was recovered, and two 10‐mL aliquots were immediately stored at 4°C. A differential count of bronchoalveolar lavage fluid (BALF) was performed on a minimum of 400 cells for allocation of treatment groups; epithelial cells were not included in the differential count.^27^ Preparation of slides was performed with 400 μL of BALF within 4 hours of sample collection, which was centrifuged using a Cytospin (90*g* for 5 minutes) and stained with modified Wright‐Giemsa stain. A differential cell count of BALF obtained on day 0 and day 14 later was performed on a minimum of 2000 cells, excluding epithelial cells.

### 2.4 DNA extraction

Total DNA was extracted from nasal swab, BALF, and transendoscopic tracheal wash (2 mL fluid) samples using a Qiagen DNEasy Tissue kit (Qiagen Inc, Mississauga, ON, Canada) as previously described.^26^ Extracted DNA was stored at −80°C until amplification and sequencing. Blank negative controls (kit only) were included in triplicate during DNA extraction.

### 2.5 EHV qPCR analysis

Primer sequences and reaction condition for EHV‐1, EHV‐2, EHV‐4, and EHV‐5 have been described previously, and reaction conditions optimized.^28^ To provide normalization, β2‐microglobulin (β2M) was used as a reference gene.^28^ Reactions were executed in duplicate, using extracted DNA from nasal swab and BALF samples as the template. Samples collected on day 0 and day 14 from the same horse were included on the same plate. Negative controls were included on each plate. A lung sample that tested positive by quantitative PCR for EHV‐1 from Ontario Veterinary College, The University of Guelph, was run in duplicate as a positive control. Cycle threshold (C_T_) values were generated using Bio‐Rad CFX Manager 3.1 software. Cycle threshold (C_T_) results <45 were classified as positive. Product was confirmed by Sanger sequencing and BLAST analysis.

### 2.6 qPCR statistical analysis

The relative expression software tool, which allows for correction for PCR efficiency and normalization with multiple reference genes, was used for analysis and has been validated previously.^29^, ^30^

### 2.7 16S and ITS amplification and sequencing

The 16S amplicon PCR forward primer (5′GTGYCAGCMGCCGCGGTAA) and reverse primer (5′GGACTACNVGGGTWTCTAAT) with forward primer barcodes were used to amplify the V4 variable region. The ITS2 amplicon PCR ITS1F forward primer (5′CTTGGTCATTTAGAGGAAGTAA) and ITS2 reverse primer (5′GCTGCGTTCTTCATCGATGC) with forward primer barcodes were used. A 30‐35 cycle PCR was performed using the HotStarTaq Plus Master Mix Kit (Qiagen) and the following conditions: 94°C for 3 minutes, followed by 30 cycles of 94°C for 30 seconds, 53°C for 40 seconds, and 72°C for 1 minute, after which a final elongation step at 72°C for 5 minutes was performed. After amplification, PCR products were checked in 2% agarose gel to determine the success of amplification and the relative intensity of bands. Amplicons then were purified using calibrated Ampure XP beads. Sequencing was performed at MR DNA (http://www.mrdnalab.com, Shallowater, Texas) on an Illumina MiSeq system using the 2 × 300 base pair paired‐end sequencing kit following the manufacturer's guidelines.

### 2.8 Sequence processing

After quality check with FastQC 11.5 and MultiQC 1.0,^31^ primers and low‐quality sequence were trimmed off the raw sequence reads using Cutadapt 1.14.^32^ The trimmed reads were used to construct amplicon sequence variants (ASVs) using dada2 1.4.0^33^ in R 3.4.1.^34^ Unless otherwise stated, all dada2 functions were used with default parameters. Reads first were filtered with dada2::filterAndTrim with a maximum expected error of 1. Error rates were learned using 2 million sequences for the forward and reverse reads separately, and these error rates were used to infer exact sequences (error correct) for each sample from dereplicated, trimmed reads. Afterward, the forward and reverse reads were merged using dada2::mergePairs. Chimeras were removed with dada2::removeBimeraDenovo, and taxonomy was assigned using the naïve Bayesian classifer^35^ as implemented in dada2::assignTaxonomy trained with the Ribosomal Database Project training set version 16. Species level assignment was done with dada2::addSpecies which uses exact matching to assign species where possible. The ASVs were aligned with ssu‐align 1.1^36^ and a phylogenetic tree constructed with FastTree 2.1.9.^37^

### 2.9 Statistical analysis

All statistical analysis was done using R 3.5.1 primarily with phyloseq 1.26.0,^38^ and vegan 2.5.3.^39^ Plots were created with ggplot2 3.1. Sequences matching mitochondria or chloroplast were removed along with any sequences that were not assigned to bacteria. Contaminant sequences present in the blank controls were removed from further analysis. A filtered copy of the ASV sequence table was created that retained ASVs present (count ≥2) in at least 2% of the samples. This served to decrease noise for downstream analysis. The full version of the sequence table was used for alpha diversity, which was assessed with the Shannon diversity index and Chao1 species richness estimator. Group means were compared using the Wilcoxon Rank Sum test (α < .05). To calculate beta diversity, the ASV counts (filtered table) were normalized by relative abundance (total‐sum scaling) and sample‐sample distance were determined using the Bray‐Curtis distance and visualized with principal coordinates analysis (PCoA).

Differentially abundant ASVs were identified using generalized linear models as implemented in DESeq2 1.22.1.^40^ Before testing, the ASV table was filtered to retain ASVs present (count ≥2) in at least 5% of the samples to remove low abundant noise. A 2 × 2 factorial model was used for testing, including an interaction term, and a *P*‐value cutoff of .05 was used to identify significant ASVs.

Normality of the distribution of the BALF differential cell counts was tested using a Shapiro‐Wilk normality test. A 2‐way repeated‐measures analysis of variance (controlling for treatment group and time point [day 0 versus day 14]) was used to assess differences in cell counts between groups. A *P*‐value ≤.05 was considered significant.

## 3 RESULTS

### 3.1 Dust concentrations

**Figure 2:**
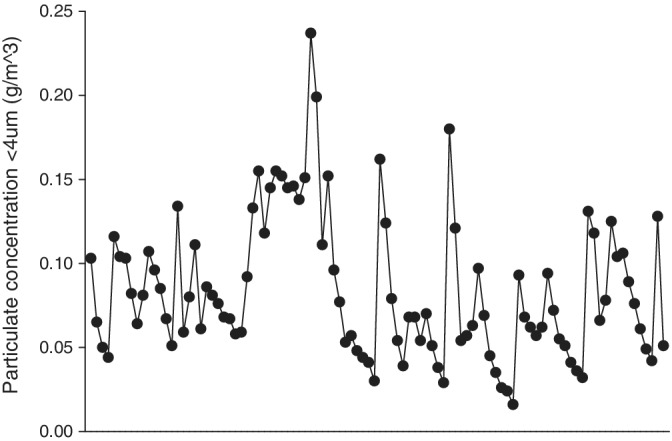
Dust concentrations of particulates <4 μm (g/m^3^) from days 6 to 14. A malfunction in the monitor resulted in the absence of data between days 1 and 5

Dust concentrations of particulates <4 μm (g/m^3^) are shown in Figure 2. A malfunction in the monitor resulted in an absence of data collection between days 1 and 5. Data collected reflected a weather event on day 9. There was a steady circadian pattern associated with husbandry (ie, stall cleaning, feeding, lunging) between 0.05 and 0.1 g/m^3^.

### 3.2 Cytology results

**Figure 3:**
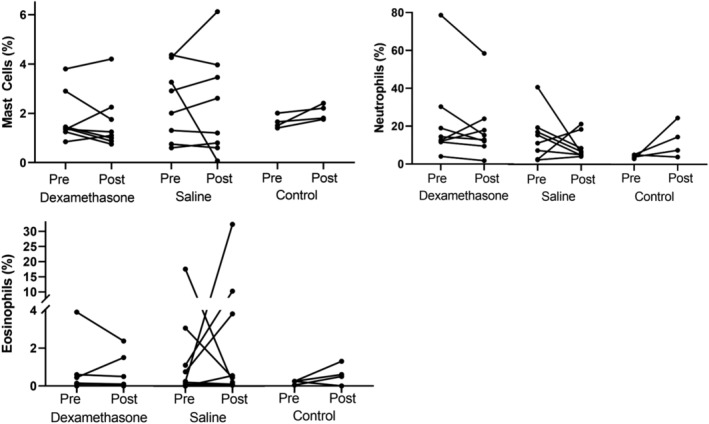
Bronchoalveolar lavage fluid differential cell counts in 16 horses with mild equine asthma before (Pre: day 0) and after (Post: day 14) treatment with nebulized dexamethasone (15 mg q24h, n = 8), or nebulized saline (3 mL q24h, n = 8). Bronchoalveolar lavage fluid differential cell counts for no treatment controls (n = 4) are provided

Bronchoalveolar lavage fluid differential cell counts for each treatment group on day 0 and day 14 are shown in Figure 3. No significant difference was found in the proportion of any cell type between groups (neutrophil, *P* = .31; eosinophil, *P* = .10; mast cell, *P* = .55; alveolar macrophage, *P* = .38; lymphocyte, *P* = .84). Furthermore, no significant difference was found in the proportion of any cell type between day 0 and day 14 (neutrophil, *P* = .91; eosinophil, *P* = .60; mast cell, *P* = .81; alveolar macrophage, *P* = .49; lymphocyte, *P* = .87). At day 14, 1 horse in Group C had normal BALF cytology, whereas the other horses had developed a mixed inflammatory phenotype of mild asthma.

### 3.3 Effect of treatment on EHV‐1,2,4,5

There was no detectable level of EHV‐1 DNA in any horse in either nasal swab or BALF samples at either day 0 or day 14 (Table 1). Changes in relative concentration of EHV‐1, 2, 4, 5 DNA are only reported below for treatment groups when detectable levels were measured at both day 0 and day 14.

**Table 1:** Number of horses in each treatment group with detectable levels of expression (Ct < 45) of EHV‐1, 2, 4, 5 in nasal swab and bronchoalveolar lavage (BAL) samples on days 0 and 14

		Day 0		Day 14	
		Nasal swab	BAL	Nasal swab	BAL
EHV‐1	Dex	0	0	0	0
	Saline	0	0	0	0
	Control	0	0	0	0
	Total	0	0	0	0
EHV‐2	Dex	3	0	7	0
	Saline	2	1	5	2
	Control	3	0	3	2
	Total	8	1	15	4
EHV‐4	Dex	5	4	5	2
	Saline	2	3	2	4
	Control	1	1	0	0
	Total	8	8	7	6
EHV‐5	Dex	4	0	0	2
	Saline	2	2	3	0
	Control	2	0	2	1
	Total	8	2	5	3

Of 20 horses, EHV‐4 was detected in nasal swab samples of 8 horses on day 0 and 7 horses on day 14 (Table 1). In BALF samples, EHV‐4 was detected in 8 horses on day 0 and 6 horses on day 14 (Table 1). Two horses had detectable EHV‐4 in both nasal swab and BALF samples on day 0 (1 treated with nebulized dexamethasone and 1 with saline), and 2 different horses had detectable EHV‐4 in both nasal swab and BALF samples on day 14 (1 treated with nebulized dexamethasone and 1 with saline). In these horses, no difference was found in the relative concentration of EHV‐4 in nasal swab samples compared to BALF samples (*P* = .56).

Three horses had detectable EHV‐4 in nasal swab samples on both day 0 and 14; all 3 were in group A (Table 1). No difference was found in relative DNA levels of EHV‐4 in response to treatment with nebulized dexamethasone at the level of the upper respiratory tract (*P* = .93), but in the lower respiratory tract, relative EHV‐4 DNA levels decreased 3.25‐fold with treatment (*P* < .001). No difference was found in relative EHV‐4 in response to treatment with nebulized saline in BALF samples (*P* = .17).

The nasal DNA levels of EHV‐2 in horses treated with nebulized dexamethasone increased by a factor of 28 (*P* = .001). Before treatment with nebulized dexamethasone, 4 horses had detectable EHV‐5 (Ct < 45); but after treatment no horses from this group had detectable DNA levels of EHV‐5 (Table 1).

In horses treated with nebulized saline, relative EHV‐2 DNA levels increased in nasal swab samples by a factor of 16 327 (*P* < .001). Only 1 horse treated with nebulized saline had detectable levels of EHV‐5 at both time points; in this horse, EHV‐5 decreased by factor of 1000 (*P* < .001). However, on day 0, 2 horses treated with nebulized saline had detectable EHV‐5, and after treatment 3 horses had very low levels of EHV‐5 (Table 1).

No change in EHV‐2 DNA level was observed in horses with no lower airway inflammation on day 0 (control group; *P* = .87), but EHV‐5 levels increased by a factor of 263 (*P* = .007).

### 3.4 Upper and lower respiratory tract microbiota

**Figure 4:**
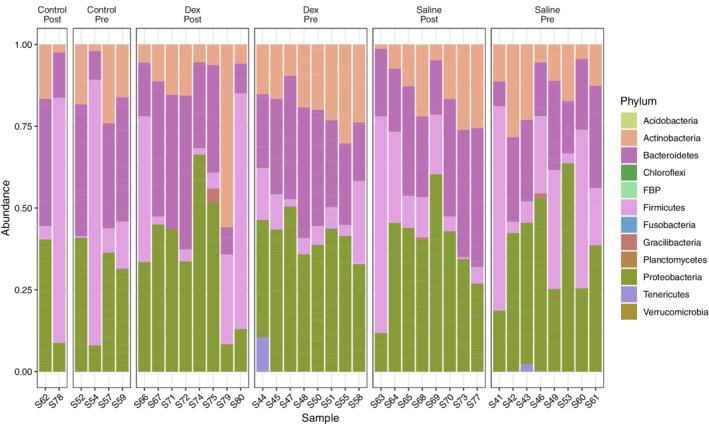
Relative abundance of microbiota phyla in the upper respiratory tract of 16 horses with mild equine asthma before (Dex_Pre: day 0) and after (Dex_Post: day 14) treatment with nebulized dexamethasone, or nebulized saline (Saline_Pre, Saline_Post). Relative abundance for no treatment controls (Control_Pre, Control_Post) are also provided

Twelve phyla were identified in nasal swab samples (Figure 4), and 16 phyla were identified in tracheal wash samples (Figure SS1), with 5 phyla showing a relative abundance >0.1% at the upper respiratory tract level: Actinobacteria, Firmicutes, Proteobacteria, Bacteroidetes, and Tenericutes (Table 2; lower respiratory tract phyla are shown in Table SS1). Four phyla represented 99.25% of the total abundance in nasal swab samples and 98.29% in tracheal wash samples: Proteobacteria, Bacteroidetes, Firmicutes, and Actinobacteria (Table 2, Table SS1, Figure 4, Figure SS1).

**Table 2:** Relative abundance of the 5 dominant microbiota phyla observed in the upper respiratory tract of horses (n = 20) over the duration of the trial, and relative abundance of genus within each phylum

Phylum (mean relative abundance, %)	Genus	Genus (mean relative abundance per phylum, %)
Proteobacteria (37.81)	*Moraxella*	11.91
	*Aureimonas*	1.51
	*Sphingomonas*	9.94
	*Pseudomonas*	9.59
	*Methylobacterium*	8.95
	*Brevundimonas*	7.88
	*Bordetella*	7.71
	*Neorhizobium*	4.99
	*Pasteurella*	3.75
	*Xylophilus*	3.57
	*Devosia*	3.12
	*Roseomonas*	2.54
	*Acinetobacter*	2.04
	*Rhizobium*	1.97
	*Actinobacillus*	1.46
	*Stenotrophomonas*	.95
	*Kingella*	.92
	*Pigmentiphaga*	.88
	*Alysiella*	.82
	*Helicobacter*	.69
	*Histophilus*	.67
	*Altererythrobacter*	.65
	*Herbaspirillum*	.62
	*Variovorax*	.50
	*Duganella*	.34
	*Thermomonas*	.33
	*Bdellovibrio*	.28
	*Alkanindiges*	.27
	*Candidatus Nasuia*	.23
	*Cystobacter*	.21
	*Ramlibacter*	.21
	*Aurantimonas*	.17
	*Polaromonas*	.13
	*Suttonella*	.12
	*Rhizobacter*	.09
	*Bosea*	.09
	*Rhodopseudomonas*	.08
	*Psychrobacter*	.08
	*Tardiphaga*	.08
	*Sphingobium*	.08
	*Haliangium*	.07
	*Massilia*	.06
	*Bradyrhizobium*	.05
	*Peredibacter*	.05
	*Xanthomonas*	.05
	*Melittangium*	.04
	*Lautropia*	.04
	*Paracoccus*	.03
	*Muribacter*	.03
	*Buchnera*	.02
	*Legionella*	.02
	*Sorangium*	.02
	*Shewanella*	.02
	*Phaselicystis*	.02
	*Enhydrobacter*	.01
	*Methylophilus*	.01
	*Acidiphilium*	.01
	*Methylopila*	.01
	*Neisseria*	.01
	*Luteibacter*	.01
Bacteroidetes (25.71)	*Hymenobacter*	64.34
	*Pedobacter*	24.64
	*Dyadobacter*	4.41
	*Bergeyella*	3.87
	*Sphingobacterium*	1.33
	*Candidatus_Sulcia*	.37
	*Spirosoma*	.36
	*Chryseobacterium*	.15
	*Porphyromonas*	.15
	*Niabella*	.11
	*Alloprevotella*	.07
	*Arcticibacter*	.06
	*Proteiniphilum*	.03
	*Taibaiella*	.03
	*Mucilaginibacter*	.02
	*Larkinella*	.02
	*Prevotella*	.02
	*Rikenellaceae*_RC9_gut_group	.01
	*Bacteroides*	.01
	*Adhaeribacter*	.01
Firmicutes (17.96)	*Staphylococcus*	75.56
	*Gemella*	7.98
	*Streptococcus*	4.93
	*Weissella*	1.79
	*Kurthia*	1.71
	*Aerococcus*	1.48
	*Jeotgalicoccus*	1.36
	*Facklamia*	1.26
	*Enterococcus*	.39
	*Granulicatella*	.38
	*Lactobacillus*	.34
	*Ruminococcaceae*_UCG‐005	.32
	*Salinicoccus*	.31
	*Planomicrobium*	.30
	*Desemzia*	.23
	*Atopostipes*	.21
	*Ignavigranum*	.18
	*Thermoactinomyces*	.15
	*Macrococcus*	.13
	*Aerosphaera*	.12
	*Oceanobacillus*	.11
	*Mogibacterium*	.08
	*Lysinibacillus*	.07
	*Solibacillus*	.07
	*Anaerococcus*	.07
	*Romboutsia*	.06
	*Trichococcus*	.06
	*Carnobacterium*	.05
	*Christensenellaceae*_R‐7_group	.05
	*Faecalitalea*	.05
	*Dolosigranulum*	.04
	*Clostridium*_sensu_stricto_1	.04
	*Ruminiclostridium*_9	.03
	*Ruminococcus*_2	.03
	*Turicibacter*	.03
	*Ruminococcaceae*_UCG‐010	.02
	*Dialister*	.02
	*Dorea*	.01
	*Negativicoccus*	.01
Actinobacteria (17.77)	*Corynebacterium*_1	24.32
	*Rathayibacter*	21.54
	*Rhodococcus*	15.42
	*Pseudarthrobacter*	5.86
	*Curtobacterium*	5.27
	*Kineococcus*	4.52
	*Aeromicrobium*	4.07
	*Rothia*	2.71
	*Kocuria*	2.55
	*Nocardioides*	2.44
	*Salana*	1.33
	*Kineosporia*	1.26
	*Nakamurella*	1.05
	*Lysinimonas*	.96
	*Patulibacter*	.92
	*Knoellia*	.83
	*Yonghaparkia*	.71
	*Brachybacterium*	.58
	*Quadrisphaera*	.45
	*Glutamicibacter*	.43
	*Brevibacterium*	.28
	*Marisediminicola*	.26
	*Solirubrobacter*	.26
	*Enteractinococcus*	.22
	*Janibacter*	.21
	*Arthrobacter*	.20
	*Sanguibacter*	.18
	*Gaiella*	.15
	*Paenarthrobacter*	.15
	*Herbiconiux*	.13
	*Ornithinimicrobium*	.11
	*Williamsia*	.11
	*Lapillicoccus*	.10
	*Dietzia*	.10
	*Promicromonospora*	.06
	*Iamia*	.06
	*Saccharopolyspora*	.05
	*Actinomycetospora*	.04
	*Haloactinobacterium*	.04
	*Frigoribacterium*	.03
	*Streptomyces*	.02
	*Bifidobacterium*	.02
	*Actinoplanes*	.01
	*Glycomyces*	.01
	*Mycobacterium*	.01
Tenericutes (.52)	*Mycoplasma*	10.00

### 3.5 Upper and lower respiratory tract mycobiota

**Figure 5:**
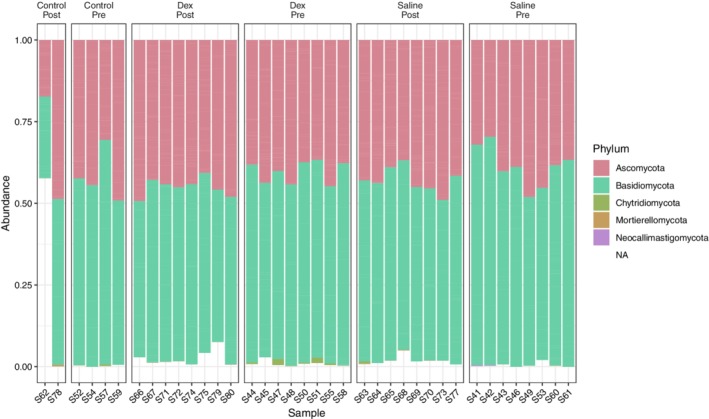
Relative abundance of mycobiota phyla in the upper respiratory tract of 16 horses with mild equine asthma before (Dex_Pre: day 0) and after (Dex_Post: day 14) treatment with nebulized dexamethasone, or nebulized saline (Saline_Pre, Saline_Post). Relative abundance for no treatment controls (Control_Pre, Control_Post) are also provided

Five phyla were identified in nasal swab samples (Figure 5), and 4 phyla were identified in tracheal wash samples (Figure SS2) with 2 phyla representing 99.78% of the total abundance in nasal swab samples and 99.42% in tracheal wash samples: Ascomycota and Basidiomycota (Table 3 and Table S2).

**Table 3:** Relative abundance of the 2 dominant mycobiota phyla observed in the upper respiratory tract of horses (n = 20) over the duration of the trial, and relative abundance of genus within each phylum

Phylum (mean relative abundance, %)	Genus	Genus (mean relative abundance per phylum, %)
Basidiomycota (56.86)	*Vishniacozyma*	33.72
	*Udeniomyces*	22.81
	*Dioszegia*	15.57
	*Filobasidium*	7.83
	*Kondoa*	4.98
	*Wallemia*	3.34
	*Bullera*	3.33
	*Bensingtonia*	1.76
	*Sporobolomyces*	1.73
	*Mrakiella*	1.38
	*Ceratobasidium*	.74
	*Cystofilobasidium*	.59
	*Leucosporidium*	.58
	*Cristinia*	.29
	*Holtermanniella*	.28
	*Coprinopsis*	.18
	*Malassezia*	.18
	*Naganishia*	.15
	*Cystobasidium*	.10
	*Chrysomyxa*	.06
	*Cryptococcus*	.06
	*Erythrobasidium*	.06
	*Papiliotrema*	.05
	*Chaetospermum*	.04
	*Tranzscheliella*	.04
	*Rhodosporidiobolus*	.02
	*Trametes*	.02
	*Genolevuria*	.02
	*Phaeotremella*	.02
	*Trechispora*	.01
	*Itersonilia*	.01
	*Symmetrospora*	.01
	*Waitea*	.01
	*Buckleyzyma*	.00
	*Serendipita*	.00
	*Efibulobasidium*	.00
Ascomycota (42.92)	*Phaeosphaeria*	17.68
	*Parastagonospora*	15.26
	*Alternaria*	14.21
	*Neoascochyta*	8.23
	*Cladosporium*	7.86
	*Chaetosphaeronema*	5.14
	*Septoriella*	3.20
	*Nigrospora*	2.42
	*Mycosphaerella*	2.41
	*Aspergillus*	2.04
	*Sarocladium*	1.71
	*Ampelomyces*	1.69
	*Ramularia*	1.48
	*Aureobasidium*	1.14
	*Articulospora*	1.13
	*Zymoseptoria*	.98
	*Helgardia*	.83
	*Tetracladium*	.81
	*Leptosphaeria*	.66
	*Gibberella*	.65
	*Radulidium*	.58
	*Paraphoma*	.55
	*Selenophoma*	.52
	*Trichopeziza*	.50
	*Trichometasphaeria*	.47
	*Arthrinium*	.46
	*Podosphaera*	.46
	*Claviceps*	.41
	*Chalastospora*	.38
	*Mycocentrospora*	.38
	*Juncaceicola*	.38
	*Pseudorobillarda*	.38
	*Acremonium*	.37
	*Stagonospora*	.36
	*Ascochyta*	.30
	*Pyrenophora*	.28
	*Torula*	.24
	*Lectera*	.23
	*Colletotrichum*	.23
	*Cistella*	.20
	*Nectriopsis*	.19
	*Paradendryphiella*	.19
	*Taphrina*	.18
	*Monographella*	.18
	*Dinemasporium*	.17
	*Hymenula*	.16
	*Protomyces*	.15
	*Rhynchosporium*	.10
	*Stemphylium*	.10
	*Oculimacula*	.10
	*Plenodomus*	.09
	*Plectosphaerella*	.08
	*Fusarium*	.08
	*Thelebolus*	.07
	*Didymella*	.07
	*Debaryomyces*	.07
	*Myrothecium*	.07
	*Periconia*	.06
	*Calycina*	.06
	*Candida*	.05
	*Dissoconium*	.05
	*Comoclathris*	.04
	*Blumeria*	.04
	*Saccharomyces*	.04
	*Ramichloridium*	.04
	*Botrytis*	.04
	*Setomelanomma*	.04
	*Knufia*	.03
	*Stachybotrys*	.03
	*Rachicladosporium*	.03
	*Apenidiella*	.03
	*Dipodascus*	.02
	*Tetrachaetum*	.02
	*Boeremia*	.02
	*Keissleriella*	.01
	*Periconiella*	.01
	*Paraphaeosphaeria*	.01
	*Penicillium*	.01
	*Dendryphion*	.01
	*Pithoascus*	.01
	*Pseudogymnoascus*	.01
	*Venturia*	.00
	*Crocicreas*	.00
	*Phaeococcomyces*	.00
	*Pyrenochaetopsis*	.00
	*Cyberlindnera*	.00
	*Preussia*	.00

### 3.6 Effects on nebulized dexamethasone and saline on the upper and lower respiratory tract environments

**Figure 6:**
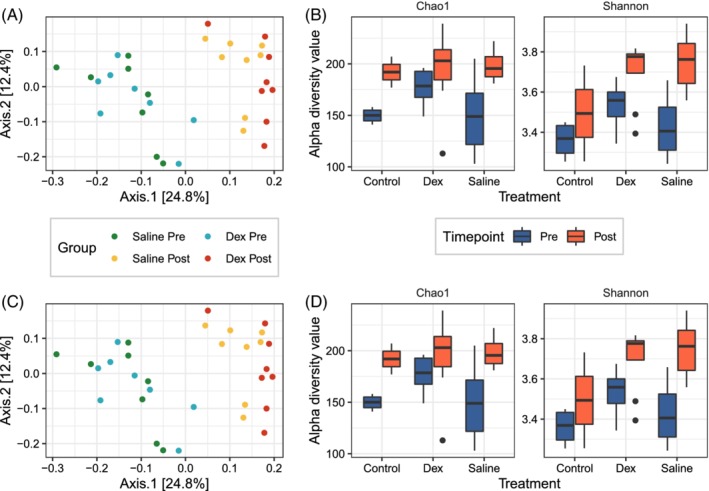
Principal coordinate analysis (PCoA) with Bray‐Curtis distance of the equine upper respiratory tract microbiota (A) and mycobiota (C). Alpha diversity measures (Chao1 and Shannon) in upper respiratory tract samples of the microbiota (B) and mycobiota (D)

Community differences with treatment at the upper respiratory tract level were visualized using PCoA and were dominated by time point effects (ie, differences between day 0 and day 14, after controlling for treatment group; the effect of sustained exposure to a dusty environment), which were more prominent than treatment effects (Figure 6A and C). In the upper respiratory tract, dexamethasone treatment resulted in a significant decrease in microbiota diversity based on Chao1 (*P* = .004) and Shannon (*P* = .004) indices (Figure 6B); saline treatment resulted in a significant increase in mycobiota diversity based on Chao1 (*P* = .04) and Shannon (*P* = .01) indices (Figure 6D). No significant differences were found in either beta or alpha diversity at the lower respiratory tract level with treatment (Figure SS3).

**Figure 7:**
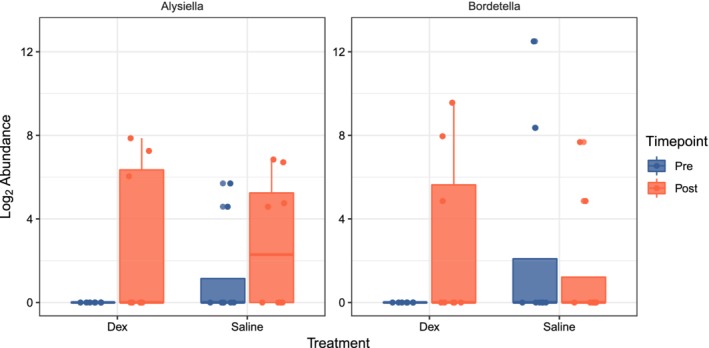
Treatment‐specific effects were tested (interaction) in the microbiota (Pre = day 0; Post = day 14) of 16 horses with mild equine asthma, treated with nebulized dexamethasone (Dex; n = 8) and nebulized saline (Saline; n = 8). Each panel shows the abundance for an individual operational taxonomic unit and is labeled with the Genus that was assigned to it

At the operational taxonomic unit (OTU) level in the microbiota, a significant change in the abundance of 8 genera was found with dexamethasone treatment (Table 4), with *Alysiella*, *Bordetella*¸ *Acinetobacter*, *Staphylococcus*, and *Pedobacter* being increased with treatment, whereas *Brevundimonas*, *Pigmentiphaga*, and an OTU assigned to the genus Corynebacterium_1 decreased with treatment. Treatment with nebulized saline had no significant effect on diversity but did alter the abundance of 3 genera, including a significant decrease in *Streptococcus* and *Brevundimonas*; *Pedobacter* was increased with treatment (Table 4). Treatment‐specific effects were tested (interaction) with 2 genera (*Alysiella* and *Bordetella*) showing a differential effect between treatments, although manual inspection of the counts for each group showed a few samples dominated by high counts suggesting a false‐positive and leading us to interpret the interaction effects with caution (Figure 7).

**Table 4:** Differential abundance associated with 14 days of nebulized dexamethasone (Dex) or saline treatment (shown as log_2_ fold−change) of individual microbiota operational taxonomic units in nasal swab samples from 16 horses with mild equine asthma

Phylum	Genus	Treatment	Log_2_ fold‐change	Standard error	*P*‐value	*P*_adj_
Bacteroidetes	*Pedobacter*	Saline	1.41	.31	4.78E‐06	.0005
Proteobacteria	*Brevundimonas*	Saline	−1.45	.37	.0001	.0054
Firmicutes	*Streptococcus*	Saline	−6.99	1.89	.0002	.0076
Proteobacteria	*Alysiella*	Dex	25.83	3.03	1.41E‐17	8.16E‐16
Proteobacteria	*Bordetella*	Dex	22.73	3.82	2.62E‐09	7.59E‐08
Bacteroidetes	*Pedobacter*	Dex	1.72	.31	2.27E‐08	4.39E‐07
Proteobacteria	*Brevundimonas*	Dex	−1.50	.38	.000083	.0012
Actinobacteria	*Corynebacterium*_1	Dex	−6.69	1.87	.00035	.0041
Proteobacteria	*Acinetobacter*	Dex	5.28	1.63	.0012	.011
Proteobacteria	*Pigmentiphaga*	Dex	−3.85	1.22	.0016	.013
Firmicutes	*Staphylococcus*	Dex	3.90	1.30	.0027	.019

The mycobiota was dominated by time point effects; when the interaction between treatment group and time point was tested, no significant genera were detected. Treatment with nebulized saline altered the abundance of 10 genera (Table 5), with *Mycocentrospora*, *Acremonium*, *Kondoa*, *Alternaria*, *Aspergillus*, *Wallemia*, and *Pseudorobillarda* increasing with treatment; *Leucosporidium*, *Septoriella*, and *Vishniacozyma* decreased with treatment. Nebulized dexamethasone treatment altered 8 genera (Table 5), with *Paradendryphiella*, *Radulidium*, *Pseudorobillarda*, *Wallemia*, and *Kondoa* increasing with treatment and *Vishniacozyma*, *Mycosphaerella*, and *Leucosporidium* decreased with treatment.

**Table 5:** Differential abundance associated with 14 days of nebulized dexamethasone (Dex) or saline treatment (shown as log_2_ fold−change) of individual mycobiota operational taxonomic units in nasal swab samples from 16 horses with mild equine asthma

Phylum	Genus	Treatment	Log_2_ fold‐change	Standard error	*P*‐value	*P*_adj_
Basidiomycota	*Vishniacozyma*	Saline	−1.20	.24	.00000005	.0000061
Basidiomycota	*Wallemia*	Saline	2.94	.62	.00000021	.00013
Ascomycota	*Septoriella*	Saline	−2.07	.45	.00000051	.00021
Ascomycota	*Mycocentrospora*	Saline	5.95	1.42	.0000026	.00065
Basidiomycota	*Leucosporidium*	Saline	−4.07	.96	.0000025	.00065
Ascomycota	*Pseudorobillarda*	Saline	3.76	.99	.00014	.0029
Ascomycota	*Acremonium*	Saline	5.07	1.38	.00023	.0041
Ascomycota	*Alternaria*	Saline	.73	.22	.0012	.019
Ascomycota	*Aspergillus*	Saline	2.86	.96	.0028	.038
Basidiomycota	*Kondoa*	Saline	.71	.25	.0038	.046
Basidiomycota	*Kondoa*	Dex	1.19	.25	.00000013	.00011
Ascomycota	*Radulidium*	Dex	3.95	.93	.0000022	.00061
Basidiomycota	*Wallemia*	Dex	2.56	.61	.0000029	.00061
Basidiomycota	*Vishniacozyma*	Dex	−1.01	.24	.0000024	.00061
Ascomycota	*Pseudorobillarda*	Dex	3.65	.96	.00015	.0022
Basidiomycota	*Leucosporidium*	Dex	−3.65	.96	.00015	.0022
Ascomycota	*Paradendryphiella*	Dex	7.12	2.40	.0031	.037
Ascomycota	*Mycosphaerella*	Dex	−1.65	.57	.004	.042

## 4 DISCUSSION

Our study describes a comprehensive investigation into the effects of 14 days of nebulized dexamethasone and saline administration in a sustained dusty environment on the bacterial, fungal, and viral communities of the respiratory tract in horses with mild asthma. Nebulized dexamethasone treatment affected the upper respiratory tract microbiota, but not the mycobiota, which was overwhelmed by the effect of a sustained dusty environment. Notably, the genus *Alternaria*, a known opportunistic pathogen and allergen in humans, was significantly increased. The equine gamma herpesviruses (EHV‐2 and ‐5) were affected by treatment, with EHV‐2 DNA increasing in response to both nebulized dexamethasone and saline, and the level of EHV‐5 decreasing. This finding highlights the importance of environmental modification as part of the treatment strategy for asthma in horses.

A strength of our study is the investigation and report of the respiratory mycobiota. The contribution of fungi to the overall community structure of the equine airways is unknown. A recent, culture‐based study investigated the prevalence of fungi in respiratory samples of horses diagnosed with mild asthma and assessed risk factors associated with the presence of fungi in the airways.^41^ These investigators obtained a positive fungal culture in 55% (402/731) horses referred for signs of poor performance or respiratory disease.^41^ Horses with fungi present in tracheal wash cytology are 2 times more likely to have mild asthma than those without.^41^ Risk factors associated with mild asthma in horses and with the presence of fungi in tracheal wash cytology included straw bedding and being fed dry hay.^41^ The most commonly isolated fungi were Penicillium (53%), Aspergillus (34%), Rhizomucor (5%), and Candida (5%).^41^ Our study was not designed to test differences between healthy horses and those with asthma, but we found that time (before versus after) had the largest effect on the mycobiota. Interestingly, only 1 of the commonly isolated fungi, *Aspergillus*, was found to increase after treatment with nebulized saline. Perhaps more importantly, the genus *Alternaria*, a known opportunistic pathogen and allergen in humans that has been increasingly recognized as a risk factor for asthma, asthma severity, and exacerbations,^42^ was significantly increased with treatment. As a major aeroallergen, prolonged exposure to *Alternaria* has been recognized as a risk factor for the development of asthma in humans.^42^

A frequent observation made after cessation of corticosteroid treatment in horses with asthma is the reemergence of clinical signs. Equine herpesviruses are ubiquitous in the equine population, establish lifelong latent infections and reactivate in times of immunosuppression or stress. It is logical to question whether the reemergence of clinical signs is a result of insufficient improvement in exposure to inhalable particulate matter, or whether corticosteroid‐derived immunosuppression leads to recrudescence of latent infection. Acute EHV‐1 and EHV‐4 infection present with mild clinical signs consistent with asthma.^20^ Equine herpesvirus‐2 and EHV‐5 are identified frequently in samples from horses with respiratory disease,^17^, ^21^ and there is some evidence that EHV‐2 is associated with poor performance and airway inflammation.^17^ In our study, we found no difference in relative expression of EHV‐1 or EHV‐4 in response to nebulized dexamethasone. Interestingly, relative expression of the equine gamma herpesviruses (EHV‐2 and EHV‐5) was affected by treatment, with EHV‐2 being upregulated in response to both nebulized dexamethasone and saline, and EHV‐5 being downregulated. To account for the potential of location to confound results (there was the potential for 1 horse to infect others in neighboring stalls), we investigated the stall locations of horses with upregulation of EHV‐2. Nebulizers also were disinfected thoroughly between horses. None of the horses that exhibited relative upregulation of EHV‐2 were next to, or across from, others; this change in expression is likely therefore a true response to nebulization. Furthermore, none of the control horses exhibited any changes in relative expression of any EHV. Nebulization of isotonic saline can relieve breathlessness (a subjective variable only assessable in humans), possibly by an increased rate of mucociliary clearance, but it does not affect lung function or increase bronchial responsiveness to bronchoprovocative challenge, allowing it to be used as a placebo.^43^, ^44^ The role of EHV‐2 in perpetuating the clinical signs of mild asthma after cessation of treatment warrants further investigation.

Many aeroallergens, including dust, endotoxin, fungi, molds, ultrafine particles, and noxious gases are found in conventional stables.^9^ Because both environment and asthmatic status have been reported to influence the equine respiratory microbiota,^45^ we elected to conduct the trial in a controlled, steady‐state environment which would differentiate between a normal, healthy response in control horses, and changes to the respiratory bacterial, fungal, and viral communities in horses with mild asthma. Because housing horses on pasture typically is considered to be an environmental improvement in coughing horses diagnosed with asthma, we did not feel pasture would be a suitable environment for our trial. Horses were exposed to the dusty stable environment for a week before initial sampling. We felt confident that all horses predisposed to developing mild asthma when exposed to suboptimal conditions would have developed airway inflammation and clinical signs of disease.^12^ Conversely, horses that retained normal BALF findings represented a healthy, ideal control group. However, even healthy horses develop transient airway neutrophilia in response to an antigenic environment (eg, organic dust challenge) which appears to be dose‐dependent.^3^, ^4^, ^5^, ^6^, ^7^ We acknowledge that the level of respirable particulates in the overall stall air does not necessarily reflect the level of dust challenge a horse experiences. Although the majority of exposure occurs in the breathing zone during feeding,^11^ it was not logistically feasible to measure the particulate concentration at the breathing zone in this trial, thus the overall stall air concentration was measured; we determined that this measurement provided additional information pertinent to the investigation.

There is strong evidence that without environmental modifications, corticosteroid treatment alone fails to normalize airway neutrophilia, even after treatment periods of up to 6 months.^46^, ^47^, ^48^, ^49^, ^50^ In a single study investigating the effects of nebulized dexamethasone in horses with asthma, a cytological improvement was reported,^24^ but there were changes to the horses' environment not reported in the study, because it was not the primary focus of the study (personal communication with the author). It is not surprising therefore that in our study no significant difference was found in the proportion of any cell type between day 0 and day 14. Furthermore, prolonged exposure to the dusty stable environment induced airway inflammation in 75% of our control horses (3/4). These findings are consistent with previous reports, in which healthy mature horses developed airway neutrophilia in response to an organic dust challenge by exposure to straw and moldy hay.^4^, ^6^, ^47^

The overwhelming influence of the environment on the disease process was observed in analysis of the respiratory mycobiota. In the microbiota, although time point effects predominated, a treatment‐specific effect was observed, with 2 genera (*Alysiella* and *Bordetella*) showing a differential effect between treatment with nebulized dexamethasone, and nebulized saline. Interestingly, the relative abundance of *Streptococcus* was not influenced by nebulized dexamethasone administration, as was observed in a trial investigating the effects of injected dexamethasone, in which the horses were kept outside.^26^ However, although treatment with nebulized saline had no significant effect on diversity, it was associated with a significant decrease in *Streptococcus* in horses with mild asthma.

Our findings highlight the importance of environmental modification as part of the treatment strategy for mild asthma in horses. Prolonged exposure to a dusty stable environment induced lower airway inflammation in healthy adult horses. The role of EHV‐2 in perpetuating clinical signs of mild asthma after completion of treatment warrants further investigation. Nebulized saline was associated with a significant decrease in *Streptococcus* in the upper respiratory tract. Nebulized dexamethasone treatment affected the upper respiratory tract microbiota, but not the mycobiota, which was overwhelmed by the effect of a sustained dusty environment.

## CONFLICT OF INTEREST DECLARATION

Authors declare no conflict of interest.

## OFF‐LABEL ANTIMICROBIAL DECLARATION

Authors declare no off‐label use of antimicrobials.

## INSTITUTIONAL ANIMAL CARE AND USE COMMITTEE (IACUC) OR OTHER APPROVAL DECLARATION

This study was conducted in accordance with the recommendations of the Canadian Council of Animal Care. The research protocol was reviewed and approved by the University of Calgary Veterinary Sciences Animal Care Committee (AC17‐0097). Informed consent was obtained from the owners of the horses enrolled in the study.

## HUMAN ETHICS APPROVAL DECLARATION

Authors declare human ethics approval was not needed for this study.

## Supporting information


**Figure S1** Relative abundance of microbiota phyla in the lower respiratory tract of 16 horses with mild equine asthma before (Dex_Pre; Day 0) and after (Dex_Post; Day 14) treatment with nebulized dexamethasone, or nebulized saline (Saline_Pre, Saline_Post). Relative abundance for no treatment controls (Control_Pre, Control_Post) are also provided.

**Figure S2** Relative abundance of mycobiota phyla in the lower respiratory tract of 16 horses with mild equine asthma before (Dex_Pre; Day 0) and after (Dex_Post; Day 14) treatment with nebulized dexamethasone, or nebulized saline (Saline_Pre, Saline_Post). Relative abundance for no treatment controls (Control_Pre, Control_Post) are also provided.

**Figure S3** Principal coordinate analysis (PCoA) with Bray‐Curtis distance of the equine lower respiratory tract microbiota (8A) and mycobiota (8C). Alpha diversity measures (Chao1 and Shannon) in lower respiratory tract samples of the microbiota (8B) and mycobiota (8D).

**Table S1** Relative abundance of the dominant microbiota phyla observed in the lower respiratory tract of horses (n = 20) over the duration of the trial, and relative abundance of genus within each phylum
**Table S2**: Relative abundance of the dominant mycobiota phyla observed in the lower respiratory tract of horses (n = 20) over the duration of the trial, and relative abundance of genus within each phylum
