# Supplementary figures and images for: Effects of nebulized dexamethasone on the respiratory microbiota and mycobiota and relative equine herpesvirus‐1, 2, 4, 5 in an equine model of asthma
Source: J Vet Intern Med. 2019 Dec 3;34(1):307–21. doi: 10.1111/jvim.15671 (PMC6979091; doi:10.1111/jvim.15671)

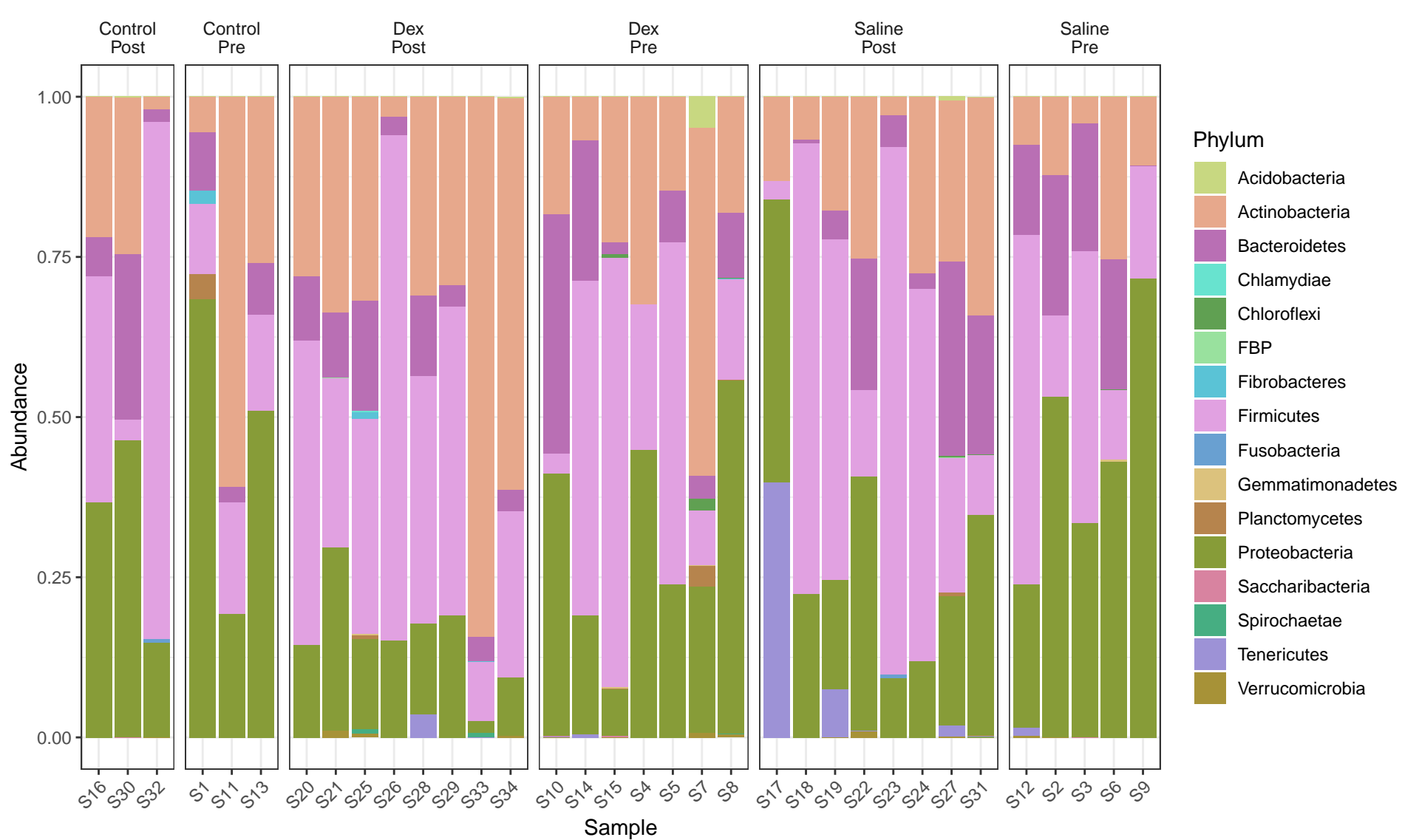

Supplement: Supplementary file 1 — Figure S1 Relative abundance of microbiota phyla in the lower respiratory tract of 16 horses with mild equine asthma before (Dex_Pre; Day 0) and after (Dex_Post; Day 14) treatment with nebulized dexamethasone, or nebulized saline (Saline_Pre, Saline_Post). Relative abundance for no treatment controls (Control_Pre, Control_Post) are also provided. [file JVIM-34-307-s001.pdf]

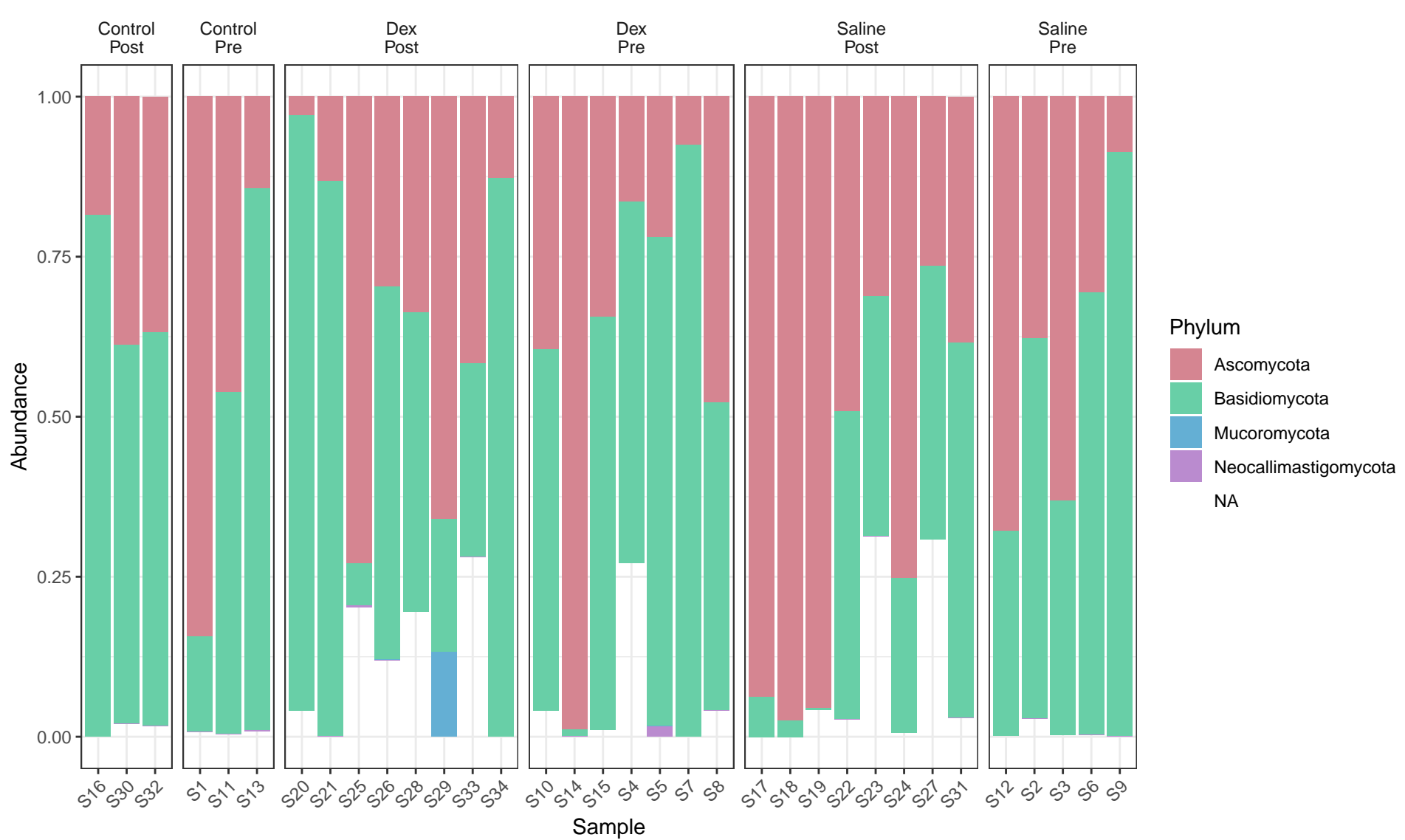

Supplement: Supplementary file 2 — Figure S2 Relative abundance of mycobiota phyla in the lower respiratory tract of 16 horses with mild equine asthma before (Dex_Pre; Day 0) and after (Dex_Post; Day 14) treatment with nebulized dexamethasone, or nebulized saline (Saline_Pre, Saline_Post). Relative abundance for no treatment controls (Control_Pre, Control_Post) are also provided. [file JVIM-34-307-s003.pdf]

A

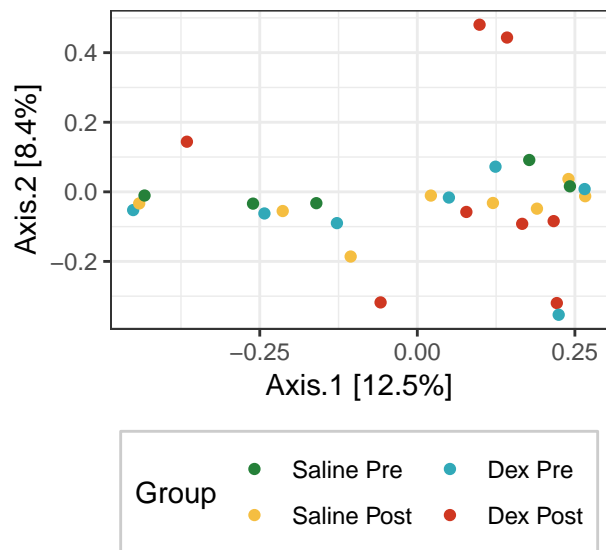

B

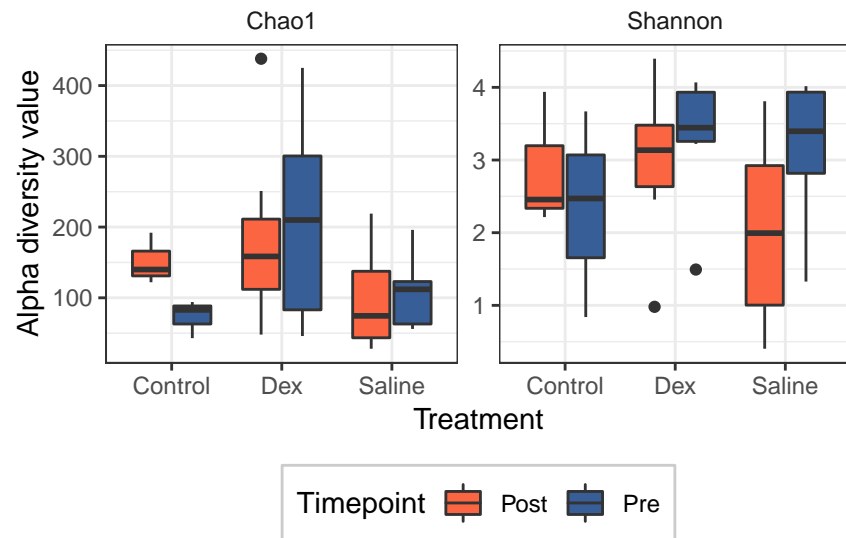

C

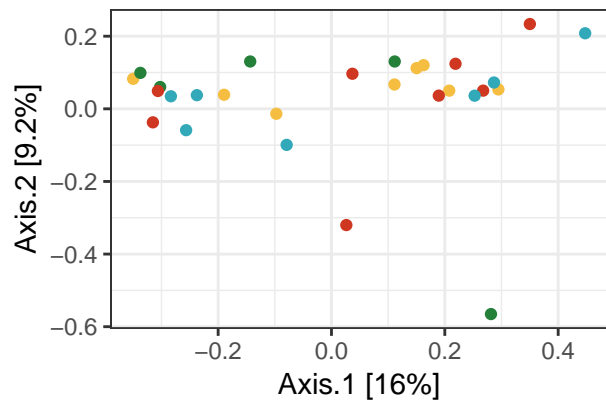

D

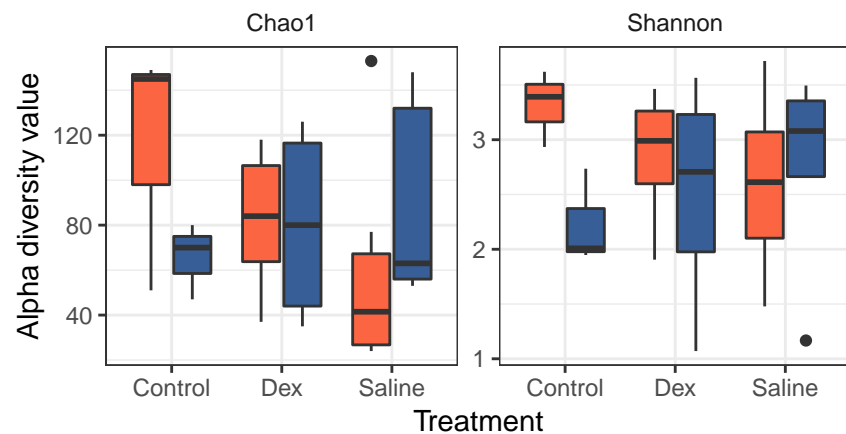

Supplement: Supplementary file 3 — Figure S3 Principal coordinate analysis (PCoA) with Bray‐Curtis distance of the equine lower respiratory tract microbiota (8A) and mycobiota (8C). Alpha diversity measures (Chao1 and Shannon) in lower respiratory tract samples of the microbiota (8B) and mycobiota (8D). [file JVIM-34-307-s004.pdf]
