# Supplementary material for: Effects of nebulized dexamethasone on the respiratory microbiota and mycobiota and relative equine herpesvirus‐1, 2, 4, 5 in an equine model of asthma
Source: J Vet Intern Med. 2019 Dec 3;34(1):307–21. doi: 10.1111/jvim.15671 (PMC6979091; doi:10.1111/jvim.15671)
Supplement: Supplementary file 4 — Table S1 Relative abundance of the dominant microbiota phyla observed in the lower respiratory tract of horses (n = 20) over the duration of the trial, and relative abundance of genus within each phylum Table S2: Relative abundance of the dominant mycobiota phyla observed in the lower respiratory tract of horses (n = 20) over the duration of the trial, and relative abundance of genus within each phylum [file JVIM-34-307-s002.pdf]

Supplementary Table 1: Relative abundance of the dominant microbiota phyla observed in the lower respiratory tract of horses (n = 20) over the duration of the trial, and relative abundance of genus within each phylum

**Phylum (mean relative abundance, %)**	**Genus**	**Genus (mean relative abundance per phylum, %)**
Actinobacteria (29.76%)	Pseudarthrobacter	45.22%
	Rhodococcus	10.56%
	Rathayibacter	9.24%
	Corynebacterium_1	8.34%
	Kitasatospora	4.36%
	Rothia	3.73%
	Curtobacterium	2.09%
	Nocardioides	1.60%
	Aeromicrobium	1.47%
	Kineococcus	1.45%
	Saccharopolyspora	1.38%
	Nocardiopsis	.95%
	Kocuria	.82%
	Nakamurella	.76%
	Brevibacterium	.60%
	Herbiconiux	.54%
	Knoellia	.41%
	Streptomyces	.38%
	Marmoricola	.34%
	Gaiella	.34%
	Williamsia	.32%
	Salana	.32%
	Patulibacter	.32%
	Dietzia	.31%
	Lapillicoccus	.29%
	Glutamicibacter	.26%
	Kineosporia	.24%
	Corynebacterium	.23%
	Solirubrobacter	.23%
	Actinomyces	.21%
	Brachybacterium	.21%
	Janibacter	.20%
	Mycobacterium	.20%
	Ornithinimicrobium	.20%
	Senegalimassilia	.18%
	CL500-29_marine_group	.16%

	Actinomycetospora	.15%
	Paenarthrobacter	.14%
	Cellulomonas	.12%
	Marisediminicola	.11%
	Flaviflexus	.11%
	Sanguibacter	.10%
	Blastococcus	.10%
	Lysinimonas	.09%
	Enteractinococcus	.09%
	Yonghaparkia	.09%
	Frigoribacterium	.07%
	Iamia	.07%
	Ornithinicoccus	.06%
	Arthrobacter	.06%
	Haloactinobacterium	.05%
	Actinomadura	.03%
	Actinoplanes	.03%
	Glycomyces	.03%
	Promicromonospora	.03%
	Bifidobacterium	.02%
Proteobacteria (28.85%)	Pseudomonas	14.83%
	Bordetella	10.78%
	Acinetobacter	10.34%
	Methylobacterium	9.25%
	Aureimonas	7.50%
	Pasteurella	5.67%
	Sphingomonas	5.61%
	Actinobacillus	4.21%
	Neorhizobium	3.43%
	Brevundimonas	2.87%
	Roseomonas	2.38%
	Acidovorax	1.78%
	Devosia	1.55%
	Enhydrobacter	1.45%
	Xylophilus	1.42%
	Janthinobacterium	1.40%
	Rhizobium	1.37%
	Alysiella	1.30%
	Muribacter	1.23%
	Brucella	1.13%
	Sphingobium	.92%
	Bdellovibrio	.86%
	Massilia	.53%

	Pigmentiphaga	.48%
	Paracoccus	.46%
	Moraxella	.42%
	Polaromonas	.41%
	Stenotrophomonas	.40%
	Lautropia	.35%
	Bradyrhizobium	.35%
	Mesorhizobium	.31%
	Kingella	.31%
	Tepidimonas	.29%
	Aurantimonas	.27%
	Altererythrobacter	.27%
	Herbaspirillum	.26%
	Neisseria	.24%
	Salmonella	.21%
	Hyphomicrobium	.19%
	Thermomonas	.18%
	Succinivibrionaceae_UCG-002	.17%
	Aquicella	.16%
	Halomonas	.16%
	Rhodobacter	.15%
	Suttonella	.14%
	Luteibacter	.13%
	Sutterella	.13%
	Ramlibacter	.12%
	Variovorax	.11%
	Tepidicella	.11%
	Steroidobacter	.10%
	Achromobacter	.10%
	Variibacter	.10%
	Tardiphaga	.09%
	Alcanivorax	.08%
	Peredibacter	.08%
	Rhodopseudomonas	.08%
	Psychrobacter	.08%
	Bosea	.07%
	Rubellimicrobium	.07%
	Aeromonas	.07%
	Methylopila	.06%
	Desulfovibrio	.06%
	Luteimonas	.06%
	Duganella	.05%
	Haliangium	.05%

	Brachymonas	.05%
	Xanthomonas	.03%
	Methylorosula	.03%
	Acidiphilium	.03%
	Alkanindiges	.03%
	Sorangium	.03%
	Nordella	.02%
	Histophilus	.02%
	Candidatus_Alysiosphaera	.01%
Firmicutes (28.42%)	Thermoactinomyces	20.44%
	Staphylococcus	15.15%
	Gemella	11.48%
	Streptococcus	8.30%
	Christensenellaceae_R-7_group	4.55%
	Lactobacillus	4.25%
	Ruminococcaceae_UCG-005	3.51%
	Anaerococcus	3.21%
	Paenisporosarcina	2.41%
	Bacillus	2.07%
	Chungangia	2.04%
	Ruminococcaceae_NK4A214_group	1.98%
	Laceyella	1.09%
	Atopostipes	1.08%
	Clostridium_sensu_stricto_1	1.02%
	Ruminococcaceae_UCG-014	1.00%
	Facklamia	.87%
	Lachnospiraceae_NK3A20_group	.85%
	Cellulosilyticum	.81%
	Jeotgalicoccus	.81%
	Roseburia	.80%
	Mogibacterium	.79%
	Ruminococcaceae_UCG-010	.69%
	Lachnospiraceae_FCS020_group	.64%
	Family_XIII_AD3011_group	.54%
	Granulicatella	.54%
	Butyrivibrio_2	.52%
	Dialister	.50%
	Aerococcus	.46%
	Planomicrobium	.38%
	Lysinibacillus	.36%
	Ruminococcus_2	.36%
	Veillonella	.34%
	Paenibacillus	.33%

	Solibacillus	.32%
	Solobacterium	.29%
	Lachnospiraceae_NK4A136_group	.29%
	Ruminococcus_1	.28%
	Saccharofermentans	.27%
	Pseudobutyrivibrio	.22%
	Oribacterium	.21%
	Enterococcus	.20%
	Dorea	.19%
	Lactococcus	.19%
	Clostridium_sensu_stricto_10	.19%
	Ruminiclostridium_5	.17%
	Ruminococcaceae_V9D2013_group	.17%
	Oscillibacter	.17%
	Lachnoclostridium_10	.15%
	Erysipelotrichaceae_UCG-004	.15%
	Paeniclostridium	.15%
	Weissella	.14%
	Blautia	.13%
	Kurthia	.12%
	Lachnoclostridium_1	.12%
	Acetitomaculum	.12%
	Turicibacter	.12%
	Lachnospiraceae_AC2044_group	.11%
	Trichococcus	.11%
	Oceanobacillus	.09%
	Ruminococcaceae_UCG-009	.09%
	Tyzzerella_3	.09%
	Lachnospiraceae_XPB1014_group	.08%
	Thermicanus	.08%
	Anaerotruncus	.07%
	Succiniclasticum	.07%
	Ruminococcaceae_UCG-013	.07%
	Carnobacterium	.07%
	Ruminococcaceae_UCG-004	.06%
	Lachnospiraceae_UCG-008	.06%
	Ruminiclostridium_6	.05%
	Clostridium_sensu_stricto_13	.05%
	Aerosphaera	.05%
	Romboutsia	.05%
	Coprococcus_1	.05%
	Ruminiclostridium_9	.05%
	Catenibacterium	.04%

	Faecalitalea	.03%
	Desemzia	.02%
	Ignavigranum	.02%
Bacteroidetes (11.26%)	Hymenobacter	58.46%
	Pedobacter	18.65%
	Rikenellaceae_RC9_gut_group	6.39%
	Proteiniphilum	3.17%
	Bergeyella	2.54%
	Prevotella_1	1.72%
	Dyadobacter	1.70%
	Bacteroides	1.44%
	Prevotellaceae_UCG-004	1.43%
	Prevotellaceae_UCG-003	1.26%
	Chryseobacterium	.56%
	Spirosoma	.55%
	Phocaeicola	.46%
	Alloprevotella	.34%
	Prevotellaceae_Ga6A1_group	.22%
	Porphyromonas	.21%
	Prevotellaceae_UCG-001	.18%
	Candidatus_Sulcia	.16%
	Vibrionimonas	.11%
	Mucilaginibacter	.09%
	Niabella	.09%
	Sphingobacterium	.09%
	Moheibacter	.06%
	Adhaeribacter	.05%
	Arcticibacter	.05%
	Prevotella	.01%
Acidobacteria (.42%)	Candidatus_Solibacter	100.00%
Planctomycetes (.32%)	Planctomyces	67.43%
	CL500-3	14.29%
	SM1A02	14.29%
	Singulisphaera	4.00%
Verrucomicrobia (.26%)	Luteolibacter	49.68%
	Chthoniobacter	33.55%
	Akkermansia	16.77%
Tenericutes (.22%)	Mycoplasma	60.77%
	Anaeroplasma	39.23%
Spirochaetae (.11%)	Treponema_2	100.00%

Supplementary Table 2: Relative abundance of the dominant mycobiota phyla observed in the lower respiratory tract of horses (n = 20) over the duration of the trial, and relative abundance of genus within each phylum

Phylum (mean relative abundance, %)	Genus	Genus (mean relative abundance per phylum, %)
Basidiomycota (56.92%)	Udeniomyces	19.64%
	Vishniacozyma	17.63%
	Trametes	17.46%
	Ustilago	7.80%
	Dioszegia	7.70%
	Filobasidium	6.30%
	Wallemia	5.76%
	Malassezia	2.32%
	Rhodotorula	2.01%
	Kondoa	1.89%
	Burgoa	1.86%
	Bullera	1.42%
	Mrakiella	1.31%
	Leucosporidium	1.25%
	Bensingtonia	1.13%
	Cristinia	1.00%
	Sporobolomyces	.87%
	Coprinopsis	.73%
	Rhodosporidiobolus	.46%
	Ceratobasidium	.36%
	Naganishia	.27%
	Cystofilobasidium	.25%
	Cryptococcus	.20%
	Holtermanniella	.15%
	Trichosporon	.14%
	Tranzscheliella	.04%
	Erythrobasidium	.02%
	Cystobasidium	.02%
	Genolevuria	.01%
	Papiliotrema	.01%
Ascomycota (42.5%)	Debaryomyces	19.37%
	Parastagonospora	15.64%
	Alternaria	14.89%
	Phaeosphaeria	11.76%
	Cladosporium	6.94%
	Candida	3.95%

	Aspergillus	3.89%
	Neoascochyta	3.54%
	Nigrospora	2.49%
	Aureobasidium	1.92%
	Chaetosphaeronema	1.84%
	Septoriella	1.73%
	Cercospora	1.37%
	Mycosphaerella	1.06%
	Saccharomyces	.97%
	Chalastospora	.85%
	Meyerozyma	.77%
	Radulidium	.77%
	Penicillium	.71%
	Cyberlindnera	.64%
	Ramularia	.61%
	Rhynchosporium	.58%
	Fusarium	.36%
	Colletotrichum	.32%
	Issatchenkia	.30%
	Claviceps	.21%
	Zymoseptoria	.20%
	Paraphoma	.19%
	Podosphaera	.14%
	Torula	.13%
	Gibberella	.13%
	Trichopeziza	.13%
	Plectosphaerella	.12%
	Sarocladium	.12%
	Helgardia	.11%
	Tetracladium	.11%
	Leptosphaeria	.10%
	Articulospora	.10%
	Dinemasporium	.07%
	Stagonospora	.07%
	Juncaceicola	.06%
	Selenophoma	.06%
	Cistella	.06%
	Trichometasphaeria	.05%
	Hymenula	.05%
	Keissleriella	.05%
	Lectera	.05%
	Calycina	.04%
	Rachicladosporium	.04%

	Stemphylium	.03%
	Myrothecium	.03%
	Plenodomus	.03%
	Monographella	.02%
	Tetrachaetum	.02%
	Ampelomyces	.02%
	Venturia	.02%
	Protomyces	.02%
	Pyrenophora	.02%
	Apenidiella	.02%
	Mycocentrospora	.02%
	Blumeria	.01%
	Taphrina	.01%
	Nectriopsis	.01%
	Crocicreas	.01%
	Arthrinium	.01%
	Pithoascus	.01%
	Dissoconium	.01%
	Pseudorobillarda	.00%
Mucoromycota (.42%)	Rhizomucor	100.00%
Neocallimastigomycota (.16%)	Neocallimastix	100.00%
